# Perceptions of Women about Menstrual Regulation Services: Qualitative Interviews from Selected Urban Areas of Dhaka

**Published:** 2007-12

**Authors:** Tanzina Nashid, Pia Olsson

**Affiliations:** 1 Ministry of Health and Family Welfare, Government of Bangladesh, Dhaka 1000, Bangladesh; 2 International Maternal and Child Health, Department of Women's and Children's Health, Uppsala University, Uppsala, Sweden

**Keywords:** Health services, Hindrances, Interviews, Menstrual regulation, Perceptions, Women, Bangladesh

## Abstract

Menstrual regulation (MR) programmes were introduced in Bangladesh in 1974 to reduce morbidity and mortality due to unsafe abortions. About 468,000 MR procedures are performed annually, and its potential is not fully used. To develop MR programmes, the voices of women could add important aspects to its acceptability. This qualitative interview study aimed to explore and describe the perceptions about MR in a sample of women from Dhaka, Bangladesh. The most prominent perception was that, despite the moral dilemma inherent in terminating pregnancies, MR was highly valued as a solution in problematic life situations. However, unprofessional attitudes and misconduct among MR providers were revealed, and there was also a lack of knowledge and openness in families. To improve the quality of MR services, professional ethics needs to be highlighted in training and supervision of providers. To improve the acceptability of MR, education on the benefits of MR has to be made available to the whole population.

## INTRODUCTION

The potential of menstrual regulation (MR) services in Bangladesh is not fully used (1–4). MR service was incorporated into the national family-planning programme in 1974 to inhibit population growth, but more importantly, to decrease the huge number of unsafe abortions, a major cause of maternal morbidity and mortality in Bangladesh (1,2,5). Since then, the Government and non-governmental organizations (NGOs) have jointly promoted MR services and training through several programmes providing training to about 12,000 health personnel (1). MR services are considered to be successful in reducing maternal mortality and hospitalization due to complications after unsafe abortion, even in remote rural areas, as the female health workers, known as Family Welfare Visitors (FWVs), provide the service (1). MR is a process of early evacuation of the uterus by manual vacuum aspiration (MVA) to regularize a delayed menstruation, irrespective of whether conception has occurred or not (3). MR is performed up to eight weeks from the last menstrual period by trained paramedics or FWVs and up to 10 weeks by physicians: this is regulated by law. Government hospitals, health centres, NGO clinics, and private facilities provide MR on an outpatient basis (2), and it should be available free of charge in government facilities (4). At present, MR is widely practised all over the country, but data on induced abortions and MR are grossly underreported (4). An estimated 468,000 MR procedures are performed annually (6). Although abortion laws are restrictive in Bangladesh, MR services are not in conflict with current abortion laws as in some other countries (2,5) and are considered culturally and politically acceptable (3).

Maternal mortality ratio is still unacceptably high—380 per 100,000 livebirths (2005), and morbidity remains a serious concern (7). Although the rate of complications from unsafe abortion has decreased over time, they are still estimated to account for one quarter of all maternal deaths (2). Existing information suggests that about 2.8% of all pregnancies are avoided through MR. A majority of these procedures are conducted in public facilities, but under unsafe conditions (1). Consequently, more than half of all admissions to gynaecology units in hospitals are due to complications after such interventions (2). Hence, the introduction of MR services in Bangladesh has not abolished complications of unsafe abortions and related maternal mortality (1). Considering this situation, the Health and Population Sector Programme (HPSP) 1998-2003 of the Ministry of Health and Family Welfare, Government of Bangladesh, included prevention of unsafe abortions through safe MR services as a key reproductive health intervention (1). At present, the Government continues the Health, Nutrition and Population Sector Programme (HNPSP) 2003-2010 to increase the availability and utilisation of user-centred, affordable, and accessible quality services and emphasizes reducing high maternal mortality ratio (8).

However, problems with MR services regarding inadequately-trained service providers, logistic support, access in rural areas, and awareness of time limits of MR have been reported (2,4), and unofficial fees, negative attitudes of service providers (4), and social stigma against MR users have been described (3).

The success of the introduction of any intervention in reproductive health largely depends on its acceptance by the people (5,9). It is reasonable to assume that perceptions and experiences of women about MR influence their willingness to seek services. Through exploring perceptions of women about MR from their own perspective, gaps in services and possible obstacles to using the services can be identified. Such information is useful for planning and implementing family-planning programmes in general and of MR in particular. This study, therefore, was designed with the aim of exploring and describing the perceptions of a sample of women in Dhaka about MR.

## MATERIALS AND METHODS

### Study design and sample

In 2005, individual interviews were conducted with 10 women from different parts of the Dhaka metropolitan area, which is characterized by modern architecture with interwoven slum areas surrounded by villages. Eighty-three percent of the population of Bangladesh is Muslim and 16% is Hindu (7). The participants of the study were selected through purposeful sampling. The criterion for selection was being a married woman aged 18-45 years. Married women were chosen because they were generally more experienced in reproductive health matters and more open to discussion about MR. To achieve maximum variation in data, women from different backgrounds and possibly different perspectives on MR were invited to participate. Efforts were made to include representatives from different ages and economic, educational and professional backgrounds. Their average age was 32 (range 18-42) years: nine were Muslim, and one was Hindu. Three women were nurses, two were university lecturers, two were housewives with higher secondary education, a third housewife had no formal education, and the remaining two were housemaids with no formal education.

### Data collection and analysis

The interviews were conducted in Bangla, the native language of the participants and interviewer. The purpose, procedure, and voluntary nature of the study were explained, and the interviews were held in private; confidentiality was secured throughout the whole research process. An interview guide with open-ended questions covering the following themes was used: awareness and understanding of MR, the performance of MR, women's experiences of MR, and engagement of partners and families in MR. The informants were encouraged to speak freely about their perceptions about MR. Probing questions were asked on what the informant said and what it meant to her. The interviews were taperecorded and lasted for 30-60 minutes, with a total of 420 minutes. The interviews were transcribed and translated into English to enable the non-Bangla-speaking co-researcher to participate in analysis. The transcribed text was analyzed by qualitative content analysis (10). All parts of the text disclosing perceptions about MR, that is meaning units, were identified, shortened, and labelled with codes. The codes were organized into categories representing a group of common perceptions.

## RESULTS

Analysis of perceptions of the women about MR resulted in nine categories (Table). They are presented following the order of their prominence in the data. Quotes from the interview transcripts are provided to illustrate the categories.

### It is a sin to destroy a foetus

When describing MR, the participants used Bangla terms, such as ‘*bachaa fela*’, meaning ‘to throw out the foetus’ and ‘*bachaa noshto kora*’, meaning ‘to destroy the foetus’. MR was perceived as a procedure performed by doctors, nurses, or persons with or without MR training, with the aim of killing the foetus. The women emphasized the necessity of confirming the pregnancy before undergoing MR. The perception about MR being a sinful act was prominent among the participants. This perception was described as being promoted by religious leaders, particularly in rural areas. A foetus has every right to be born, and it is not right to kill the foetus, whether male or female, as this was considered an act against religion and humanity. Even to discuss MR, as in the interview, was perceived as a sin. In later pregnancy, MR was considered to be more offensive, and the women felt guiltier, indicating that the difference between MR and abortion was unclear to some women.

It's a life; the life comes in it slowly. So, I think that it's a contemptible sin. If one does not want to have baby, then it's better not to conceive …. MR is very bad; it is done within three months. If 10 months have passed, then a whole human has developed. Whether it will be a male or a female child, it is a life, and I think it is a sin.

**Table:** Categories of perceptions of women about MR

1. It is a sin to destroy a foetus
2. Contraceptives are preferred over MR
3. MR is justifiable in difficult life situations
4. A relief from social embarrassment
5. Safe compared to traditional clandestine procedures
6. Dangerous in late pregnancy and in unskilled hands
7. Some MR providers disrespect and exploit patients
8. Hiding MR from family and society is common
9. Increase knowledge and openness towards MR

MR=Menstrual regulation

### Contraceptives are preferred over MR

The participants favoured the use of contraceptives as a means of avoiding the sin and hazards associated with MR. However, one problem was outlined: husbands usually decided on contraceptives. Typically, men were not interested in using condoms and did not allow their wives to use contraceptives. The participants were disappointed with attitudes of their husbands and wanted them to accept contraceptives. A predominant perception was that women should not depend on their husbands regarding contraceptives, but be encouraged to take more responsibility for using family-planning methods, thereby, avoiding unwanted pregnancies and MR, as it is the woman who suffers the burden of rearing and caring for the child.

The husband should be made to understand. Especially, the husbands, in most cases, do not understand. The husbands usually do not agree to use any contraceptives. But they should be motivated …. The wife will make the husband understand that.

The participants also mentioned contraceptive failure as a reason for choosing MR. Besides, broken condoms, forgetting to take oral pills, and inadequate knowledge about the lactational amenorrhea method (LAM) period were also cited as reasons for MR.

### MR is justifiable in difficult life situations

Unwanted pregnancies were considered to be a problem, an accident, and a mistake in a woman's life. A very high price could be paid for such a situation in which the pregnancy was continued and the baby born. Rearing a child in these hard times was considered to be difficult, with high prices and all attention needed to bring up the child properly. MR was considered to be a ‘relief’ and ‘freedom’ in a woman's life that enabled her to get rid of the ‘trouble’. The participants wanted MR as an option in the case of a mistimed pregnancy.

It (pregnancy) may happen by mistake, and humans make mistakes. This is not intentional, and if it was within their will, then they did not want to destroy it. For any reason, may be by mistake, it can happen. Everybody can make mistake and then they do MR.

Justifiable reasons mentioned for MR were: limitation of family size, economic hardship, child spacing, continuation of women studies or career in the city, disharmony in married life, and women were engaged in prostitution. Undergoing MR in the case of a pregnancy out of wedlock was perceived as both a possibility and a disrespectful act (*‘jaurami’*). MR was also described as being used for sex selection, when a female foetus was diagnosed in early pregnancy, and the desire was to have a boy. Perceptions of women about sex selection varied from acceptance to dissociation.

### A relief from social embarrassment

MR was viewed as a relief from social embarrassment, particularly for unmarried and older married women. Many young unmarried pregnant girls were said to commit suicide due to the fear of being shunned by society and being ashamed of a pregnancy out of wedlock. Older women who became pregnant after earlier children had married and when they had grand children were criticized and looked down upon by society. In such situations, MR was a relief from shame.

The benefit (of MR), relief from shame … there are such families where the women conceive in the older age, she has daughter-in-law, has a grand child; then she could not continue the pregnancy because of shame.

### Safe compared to traditional clandestine procedures

The participants described some dangerous, unhygienic, clandestine procedures, which were practised and caused maternal death, and they mentioned the use of abortion sticks and roots of herbs that were inserted through the birth canal and caused much suffering and often death for the woman.

Then there is another way to extract the foetus … with the roots of the tree … that was cut and then put on the passage of urine with a piece of string. It works strongly, and it is dangerous. In our area, two women died in this way, two women died ….

The women did not support such procedures but preferred the MR to be done by health professionals, resulting in less sufferings. However, the places where MR was done depended on the ability of women to pay: those who can afford it preferred MR to be performed in a clinic by a gynaecologist, a doctor, or a provider who was experienced and had a good reputation.

### Dangerous in late pregnancy and in unskilled hands

The participants considered MR a relatively safe procedure within the first three months of pregnancy; however, they did not trust all providers. Besides hospitals, clinics, and health centres, MR was performed secretly at the residence of providers who did not keep records. Unskilled persons should be avoided as they conduct MR in a hurry, to get more profit, and cause pain, bleeding, and infection. Other harmful effects of MR mentioned were cancer, perforation, prolapsed uterus, and white discharge. Before having an MR, a woman must take into consideration these harmful effects.

MR providers without professional training were ‘*ayas*’ (nurse maids) or birth attendants who had learnt to do MR by observing the practices of nurses and doctors. They sometimes performed MR in late pregnancy, even in the sixth month and let the patient suffer. The women described some special procedures that were used in the later part of pregnancy, such as cutting the foetus into different pieces and removing those along with the syringe. These procedures were more difficult and dangerous for the women but were considered as MR by the informants.

There are many women who can or cannot, they perform that. (…) When the pregnancy passed four months, they just opened the mouth (of the uterus). I saw the women of my area had experienced that, my sister had experienced that …. My sister delivered the baby; it was the size of the baby of a rat, which fell out after seven days.

### Some MR providers disrespect and exploit patients

The providers took advantage of the stigma attached to a mistimed pregnancy and having an MR, embarrassed them, and charged more than was stipulated. The cost of MR varied, and some providers were greedier than others. Although the government hospitals and health centres are supposed to provide the services free of charge, they often charged Tk 100-200 (1 US$=approximately Tk 70) depending on how advanced the pregnancy was: necessary drugs were also not always available, and patients could be charged up to Tk 2,000 for these. NGOs and population programmes generally charged less, whereas private clinics could charge Tk 5,000-12,000.

Women in difficult situations, such as unmarried young girls and women in advanced stages of pregnancy, were often charged more. Sometimes, the providers embarrassed the women and accompanying parents by questioning their marital status and demanding consent from their husband. The providers also scolded young patients if they did not tolerate the pain when undergoing MR, and extra fees were demanded for the management of pain.

They work hiding. Hiding, in their residence, they tell clients to come after sunset and not to tell any body. They have luxurious house, and they earn a lot. They can identify the girls who are pregnant without marriage, and they demand a lot of money from them …. I have done that …. Though it was two months pregnancy, she put her hand on my abdomen and said that the foetus was moving; she said so to take more money from me …. Then she said, tell me how much money you will give me or you will have much pain. They said like this; they are greedy.

### Hiding MR from family and society is common

The participants perceived that, due to strong stigma attached to MR, those who had MR seldom disclosed it to their family members and society. Older family members and religious leaders were mostly against MR and were, therefore, not informed. A few confidants, such as husbands and close relatives, could be told. Due to strong stigma against pregnancy out of wedlock, families could force unmarried mothers to do an MR so as to prevent social exclusion and difficulties in finding a future husband.

The husband usually takes the decision, whether his wife wants to continue it or not. But in the case of illegal relationship in many cases whether she is literate or illiterate, she took her own decision. She took the decision under pressure of the society as there is no alternative way without this (MR). She does not wait for the decision of the male.

### Increase knowledge and openness towards MR

Openness in discussing the matter, and tolerance towards those using this opportunity, was demanded by the informants. They wanted more information and knowledge regarding reproductive matters, including MR. There were various locations in the country where no MR facility is available, and the people of those regions lacked information about MR. The importance of increasing knowledge about MR among the deprived sections of the society was emphasized. The women also wanted family members to be more open-minded.

I think that our mind should be broader so that our society can accept this (MR), and no resistance should come from our families.

## DISCUSSION

The transferability of the results from this qualitative study to other settings is limited, as the results were based on interviews about MR with a small sample of women in urban Dhaka where access to MR services is relatively good. Nevertheless, the study provided information on perceptions about MR, which generate ideas for improvement of the MR programme.

### MR is needed for mistimed pregnancies

While recognizing the moral dilemma inherent in terminating pregnancies, the study women perceived MR as important for mistimed pregnancies, prevention of unsafe abortions, and social embarrassment. MR was appreciated as a solution in exceptionally problematic situations. As in most cultures, terminations of pregnancy are accompanied by feelings of ambivalence, guilt, relief, and gratefulness (11).

The study women disapproved of premarital pregnancies which corroborates the findings of other studies from Bangladesh (12,13). However, despite the societal norms discouraging their existence (13), a substantial proportion of the adult population has extramarital sexual activity, including commercial relationships. A pregnancy out of wedlock, or one which the family does not recognize within a marriage or which results from sexual violence, is frequently terminated by either clandestine abortion or MR (12,13). The ignorance of reproductive issues and MR was also demonstrated in a recent study (14), in which 57% of unmarried and over 40% of married adolescents had never heard of MR, and over half of married adolescents were unaware of the consequences of unprotected sex. As many adolescents do not have the knowledge needed for recognizing early pregnancy, they are rejected by MR clinics because their pregnancy is too far advanced (15) and are more likely to seek second trimester abortions than older women (12). Thus, the provision of education on contraception, consequence of unprotected sex, signs of early pregnancy, and information on MR for all boys and girls should be provided in an adolescent-friendly way (14,15).

Pregnancy in older women is considered to be shameful, and MR could be an acceptable reliever of this shame according to the informants. Similar perceptions have been described in some African and Indian cultures (16). Fertility of grandmothers is lower compared to that of non-grandmothers, but MR and abortion rates among older women are higher (16). To reduce unwanted pregnancies for this age-group, the importance of contraceptive-use for older women needs to be included in the information available to the public and the training of health staff to reduce unwanted pregnancies for this age-group.

### Family support

The decision to have an MR is not easy, especially when the woman is from a traditional society. No matter how important the cause was for the woman and how hard it would be for her to continue the pregnancy, family members, especially the elders, expressed strong opinions against MR. Dominancy of males in Bangladeshi society is reflected in who decides on contraceptive-use, the need for obtaining consent from the husband to use contraceptives and to do an MR. As women need to be accompanied when they go to the hospital, lack of support makes them vulnerable to clandestine and unsafe procedures. Support from the family would help them to do MR in a hospital, and it has been shown that the introduction of MR in local tertiary hospitals decreases the number of hospital admissions after unsafe abortions (17). Steps need to be taken to sensitize communities about MR and its role in improving reproductive health and any impact on family health. The creation of women's support groups and long-term strategies for developing increased openness on reproductive issues should be promoted as a means for facilitating family support.

### Unprofessional management of MR

One hindrance to the use of MR was the presence of unskilled, greedy, and insulting providers in some facilities. Non-authorized providers and providers not following the regulations were a serious problem. Similar problems have also been reported in other South Asia countries, such as India and Nepal (2,18). Non-adherence to the time limitation of MR and incomplete conduction of MR were frequently followed by complications that women were aware of and which made them hesitant to seek MR. The lack of drugs in the health facilities increased costs for women, as they had to buy analgesics and antibiotics from outside stores. In addition to the sufferings of patients, it is suggested that pain intolerance can lead to an increased incidence of retained products of conception (5,19). The misconduct of MR providers has to be addressed, as do the mechanisms for patients receiving necessary drugs. The identification of female foetuses for gender selection by MR providers was also mentioned in the interviews. The dynamics of son-preference and gender inequality leading to the increased use of sophisticated technology for the practice of sex determination followed by pregnancy termination was also reported in India and China (20). Increased efforts are, thus, needed to abolish the unethical practice that violates human rights.

Unprofessional attitudes and misconduct by MR providers towards their patients, particularly towards young unmarried women, were reported during the interview. This phenomenon is common in several other countries (21). The present study revealed that some providers took advantage of the social stigma and demanded extra money from the patients. The participants also complained of providers in the government facilities charging high fees, where the MR services should be free of charge: the low-income level of the people forces them to undergo cheaper and often unsafe procedures elsewhere (4). Measures need to be taken for dealing with the problems that constitute a major hindrance to safe MR services.

Further studies are needed to embrace the perceptions about MR among different groups of women and family members and among urban and rural MR providers. Data triangulation, including surveys, would further widen the understanding of how MR is perceived and strengthen the evidence for use of MR.
